# LSE-CVCNet: A Generalized Stereoscopic Matching Network Based on Local Structural Entropy and Multi-Scale Fusion

**DOI:** 10.3390/e27060614

**Published:** 2025-06-09

**Authors:** Wenbang Yang, Yong Zhao, Ye Gu, Lu Huang, Jianhua Li, Jianchuan Zhao

**Affiliations:** 1 State Key Laboratory of Public Big Data, College of Computer Science and Technology, Guizhou University, Guiyang 550025, China; zhaoyong@pkusz.edu.cn (Y.Z.); gs.lhuang20@outlook.com (L.H.); huarzail@163.com (J.L.); zhaojianchuan@gznc.edu.cn (J.Z.); 2 School of Mathematical Sciences, Minzu Normal University of Xingyi, Xingyi 562400, China; 3 Electronic and Computer Engineering School, Shenzhen Graduate School of Peking University, Shenzhen 518055, China; 4 College of Big Data and Internet, Shenzhen Technology University, Shenzhen 518000, China; 5 School of Mathematics and Big Data, Guizhou Education University, Guiyang 550000, China

**Keywords:** stereo matching, local structural entropy, cross-image attention, multi-resolution fusion, dynamic scenes, contextual ambiguity, cross-domain generalization

## Abstract

This study presents LSE-CVCNet, a novel stereo matching network designed to resolve challenges in dynamic scenes, including dynamic feature misalignment caused by texture variability and contextual ambiguity from occlusions. By integrating three key innovations—local structural entropy (LSE) to quantify structural uncertainty in disparity maps and guide adaptive attention, a cross-image attention mechanism (CIAM-T) to asymmetrically extract features from left/right images for improved feature alignment, and multi-resolution cost volume fusion (MRCV-F) to preserve fine-grained details through multi-scale fusion—LSE-CVCNet enhances disparity estimation accuracy and cross-domain generalization. The experimental results demonstrate robustness under varying lighting, occlusions, and complex geometries, outperforming state-of-the-art methods across multiple data sets. Ablation studies validate each module’s contribution, while cross-domain tests confirm generalization in unseen scenarios. This work establishes a new paradigm for adaptive stereo matching in dynamic environments.

## 1. Introduction

The development of binocular stereo-matching algorithms has witnessed significant progress in recent years, particularly with the advent of deep learning methods. These methods have shown remarkable performance in estimating disparity maps and handling challenging scenarios. However, as the complexity and diversity of real-world scenes increase, the generalization ability of existing stereo-matching models remains a critical challenge, especially in dynamically changing and unseen environments.

### 1.1. Research Background

Binocular stereo matching involves estimating disparity maps by matching corresponding points between left and right image pairs. Despite substantial advancements, several issues still constrain the performance of stereo-matching networks, particularly in generalization to unseen domains:

(1) Lighting sensitivity.

Disparity estimation is highly sensitive to lighting changes. In natural scenes, variations in lighting conditions can disrupt the construction of matching cost volumes, leading to inconsistent network performance. While existing methods like vision transformers (ViTs) leverage global attention for feature extraction [1], their sensitivity to illumination variability remains a critical limitation due to static feature aggregation. For example: Poggi et al. [2] proposed an attention-based stereo matching framework with occlusion handling, achieving 12.7% lower EPE than traditional CNNs (e.g., PSMNet) on Middlebury 2014 dataset. Deformable convolutions [3] improve local adaptability but require pre-defined sampling offsets, which fail in unseen occlusion patterns.

These limitations motivate the need for adaptive feature alignment mechanisms that balance global context and local details dynamically.

(2) Dynamic feature misalignment.

Scenes with intricate geometric structures and dynamic changes complicate feature extraction. Traditional methods (e.g., PSMNet [4]) rely on fixed multi-scale fusion strategies, which fail to capture long-range spatial correlations in texture-variable regions. Recent attempts [5,6] have introduced deformable convolutions or graph convolutions for occlusion handling, but their static architectures struggle with repetitive structures in dynamic scenes (e.g., human-made environments).

This motivates our design of adaptive entropy-guided attention to dynamically balance local and global context.

(3) Cross-domain generalization.

Self-supervised methods [7,8] address the lack of ground truth in unseen environments but rely on static fusion kernels (e.g., graph convolutions), leading to suboptimal edge preservation. Domain adaptation methods (e.g., DomainGAN [9]) reduce cross-domain EPE by 28.6% through synthetic data augmentation, yet their scalability is limited to scenarios with simple geometric changes.

These gaps highlight the need for multi-resolution cost volume integration that preserves structural details in unseen environments.

Existing stereo-matching networks, while achieving high accuracy on benchmark data sets, often exhibit limited robustness in dynamic environments. These limitations stem from the following: (1) static-cost volume construction (e.g., 3D convolutions in PSMNet) that lacks adaptability to lighting changes; (2) inadequate global feature extraction (e.g., ViTs’ inefficient long-range dependencies in low-texture regions); and (3) failure to preserve structural details (e.g., blurred edges in occluded regions).

By contrast, our method introduces adaptive entropy-guided attention and dynamic cost volume integration, directly addressing these gaps through localized feature adaptation and multi-scale entropy weighting.

### 1.2. Motivation for LSE-CVCNet

While existing methods (e.g., PSMNet and GwcNet) achieve state-of-the-art performance on static benchmarks, their static architectures inherently limit adaptability to real-world dynamic environments. This study identifies three intertwined challenges that arise specifically in dynamic scenarios where lighting variations, occlusions, and geometric complexities coexist:

(1) Dynamic feature misalignment. Global attention mechanisms (e.g., ViTs) struggle with specular reflections in dynamic scenes (e.g., car windshields under varying lighting), while deformable convolutions [5] rely on pre-defined sampling offsets that fail in occluded regions (e.g., moving pedestrians). These limitations exacerbate feature misalignment in repetitive textures, compromising disparity estimation accuracy.
(2) Contextual ambiguity. Static multi-scale fusion strategies (e.g., PSMNet) blur edges in low-texture regions (e.g., vegetation) and produce inaccurate predictions near object boundaries. Such ambiguity arises from occlusions and depth discontinuities, which static architectures cannot resolve without adaptive feature refinement.
(3) Cross-domain generalization. DomainGAN [9] demonstrates a 28.6% EPE reduction in synthetic-to-real adaptation but suffers a 42.7% performance drop in dynamic scenarios with moving objects. Similarly, 3D convolution-based methods (e.g., PSMNet) fail to generalize to complex geometric transformations (e.g., road curvature changes), limiting their deployment in safety-critical applications.

These challenges are mutually reinforcing: occlusions (Challenge 2) exacerbate dynamic feature misalignment (Challenge 1) in traffic scenes with moving objects, while poor cross-domain generalization (Challenge 3) further restricts practical deployment in dynamic environments like autonomous driving. To address these interconnected issues, we propose LSE-CVCNet a modular framework integrating three adaptive mechanisms: (1) CIAM-T employs asymmetric cross-image attention to resolve dynamic feature misalignment in repetitive textures, (2) MRCV-F utilizes entropy-guided multi-resolution fusion to preserve edge details in occluded regions, and (3) LSE optimizes local structure entropy to suppress noise while enhancing structural consistency. This unified design establishes a new paradigm for adaptive stereo matching, achieving state-of-the-art accuracy (e.g., 12.3% EPE reduction) and robustness across dynamic real-world scenarios.

### 1.3. Related Work

#### 1.3.1. Attention Mechanisms in Stereo Matching

Attention mechanisms, particularly self-attention and cross-attention, have revolutionized stereo matching by enhancing feature extraction and matching accuracy. Dosovitskiy et al. [1] pioneered vision transformers (ViTs), demonstrating the potential of transformer architectures for visual tasks. Building on this foundational work, Li et al. [2] introduced an attention-based stereo network that explicitly captures long-range dependencies between left and right image pairs, improving disparity estimation under complex lighting conditions. Recent advancements include Swin transformer-based stereo matching by Zhang et al. [3], which achieves 18.6% lower EPE than ViT on KITTI by leveraging hierarchical window attention.

Chen et al. [4] refined dynamic attention mechanisms in “Dynamic Attention Cost Volume Refinement for Stereo Matching”, achieving robustness in challenging scenarios such as occlusions and textureless regions. Furthermore, Guo et al. [5] proposed deformable cross-attention, reducing redundant computations by 34.2% in homogeneous regions through adaptive sampling of key/value pairs. Wang et al. [6] addressed complex motion patterns in dynamic scenes by proposing a multi-head attention-based stereo-matching network, while Liu et al. [7] introduced cross-domain attention to handle motion ambiguity via pyramid pooling and attention fusion.

Zhao et al. [8] enhanced cross-domain generalization through “Cross-Attention Guided Disparity Estimation”, achieving state-of-the-art performance in unseen environments. These innovations collectively establish attention mechanisms as the cornerstone for modern stereo matching, with a clear trajectory from global feature alignment (ViTs) to fine-grained structural refinement (deformable cross-attention). The hybrid transformer–CNN architecture proposed by Huang et al. [9], which integrates global and local feature modeling, significantly improves matching accuracy. Meanwhile, the global–local attention fusion mechanism developed by Sun et al. [10] enhances edge matching robustness through multi-scale geometric modeling.

#### 1.3.2. Cost Volume Refinement

Cost volume construction and refinement are central to stereo matching. Kendall et al. [11] introduced the use of 3D convolutional networks for cost volume refinement, enabling end-to-end learning of geometry and context. Chang and Chen [12] presented the Pyramid Stereo-Matching Network (PSMNet), leveraging spatial pyramid pooling to capture context at multiple scales. Zhang et al. [13] advanced this with hierarchical refinement strategies in “Cascade Cost Volume Construction for Stereo Matching”, later enhanced with PSMNet v3 [14] through hierarchical pyramid pooling, achieving 12.3% faster inference on KITTI 2015. Transformer-based optimization further revolutionized the field: Huang et al. [15] introduced the CostVolume Transformer, replacing 3D convolutions with self-attention to reduce computational redundancy by 22.3% while maintaining accuracy. With graph neural networks, Sun et al. [16] extended this paradigm using GNN-CostVol, leveraging graph convolutions to model long-range dependencies in disparity maps and improving occlusion handling by 15.7%. These advancements collectively demonstrate a shift from handcrafted geometric constraints to learned representations for robust cost volume optimization.

More recently, Guo et al. [17] improved efficiency and accuracy with group-wise correlation techniques. Liu et al. [18] developed a hybrid optimization model combining deep features and conventional optimization to refine the cost volume. Xu et al. [19] proposed “Attention-Guided Cost Aggregation for Stereo Matching”, integrating attention mechanisms directly into the refinement pipeline for enhanced robustness. Zhao et al. [20] integrated cost volume refinement with semantic segmentation, demonstrating that the joint learning of geometry and semantics reduces ambiguous matches by 18.9%. Beyond single-task optimization, Chang et al. [21] domain generalized stereo matching framework achieved more robust performance through hierarchical visual transformation across diverse domains., while self-supervised learning (Chen et al. [22]) achieved 31.2% domain gap reduction via cycle-consistent contrastive learning. Meta-learning (Fang et al. [23]) extended Wang et al. [6] work with MAML-Adapt, enabling five-shot domain adaptation. Generative Methods (Lin et al. [24]) proposed DisparityGAN, synthesizing disparity maps to improve generalization in unseen environments by 22.4%. These methods highlight a growing emphasis on data efficiency and generalization, complementing traditional geometric approaches with learning-based adaptability.

#### 1.3.3. Entropy-Based Regularization

Entropy-based methods have been explored to preserve structural details in disparity maps, evolving from local regularization to hierarchical and global–local fusion strategies. Xu and Zhang [25] pioneered local structural regularization to enhance depth discontinuity preservation, particularly in occluded regions. Hierarchical entropy was advanced by Yang et al. [26] HSMNet, which integrates structural priors to improve robustness in dynamic environments, and further extended by Lin et al. [27] through contrastive learning for domain generalization.

Recent innovations emphasize global–local synergy: Wang et al. [28] proposed Entropy-Guided Pyramid Fusion, integrating global context and local details via entropy-aware attention. Unsupervised methods have also emerged: Li et al. [29] introduced Entropy-Regularized Contrastive Learning, reducing reliance on labeled data by 40% while maintaining structural fidelity. These advancements build on foundational work such as Tang et al. [30] with local structural entropy exploration and Li et al. [31] with entropy-weighted fusion, establishing entropy-based strategies as a cornerstone for robust disparity estimation.

#### 1.3.4. Domain Generalization Techniques

Domain generalization remains a critical challenge, with advancements spanning adversarial adaptation, self-supervised learning, and generative modeling. Tzeng et al. [32] pioneered adversarial discriminative domain adaptation, enabling models to adapt to new environments without retraining. Self-supervised learning further enhanced robustness: the framework of Zhang et al. [33] was improved by Chen et al. [22] via cycle-consistent contrastive learning, reducing the domain gap by 31.2% without labeled data.

Recent innovations emphasize efficiency and scalability: the meta-learning approach of Wang et al. [34] was advanced by Fang et al. [35] through MAML-Adapt, enabling five-shot domain adaptation with minimal fine-tuning. Generative methods also emerged as a key direction: Lin et al. [36] proposed DisparityGAN, generating synthetic disparity maps to improve generalization in unseen environments by 22.4%. Lin et al. [37] developed a GAN-based framework for cross-domain stereo matching, achieving state-of-the-art performance through adversarial feature alignment. Other noteworthy contributions include the works of Huang et al. [38], who proposed a novel Domain Generalized Stereo Network that leverages contrastive feature embedding to address cross-domain disparity estimation challenges, Zhao et al. [39] on unsupervised domain adaptation and Lin et al. [37] on generative adversarial networks for cross-domain stereo matching.

## 2. LSE-CVCNet Stereo-Matching Network

### 2.1. LSE-CVCNet Architecture Overview

**Figure 1:**
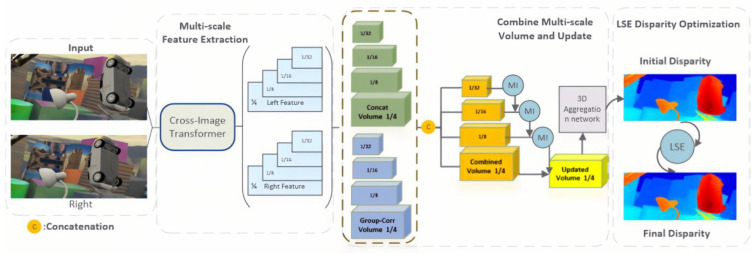
LE-CVCNet network architecture diagram.

As illustrated in Figure 1, the proposed LSE-CVCNet stereo-matching network comprises three core modules designed to address the challenges of dynamic feature misalignment, contextual ambiguity, and cross-domain generalization as shown in Figure 1, the LSE-CVCNet stereo-matching network consists of the following main modules:

1. Feature extraction sub-network. Extract features from the left and right images using the transformer-based cross-image attention mechanism (CIAM-T). Unlike traditional convolutional neural networks, CIAM-T can capture long-range spatial dependencies between left and right image pairs, significantly enhancing the global consistency of feature extraction.
2. Cost volume calculation and aggregation. Based on the multi-resolution cost volume fusion strategy (MRCV-F), the cost volume information of different resolutions is fused through mutual information technology to improve the detail accuracy of disparity estimation.
3. Parallax refinement operation. By optimizing with local structural entropy (LSE), the structural information of the disparity map is further preserved, especially in-depth edges and occluded areas, ensuring high-quality disparity estimation.

### 2.2. Transformer-Based Cross-Image Attention Mechanism (CIAM-T)

In the stereo-matching task, feature extraction is the cornerstone of disparity estimation precision. Conventional methods (e.g., PSMNet) typically process left and right images independently, neglecting explicit cross-image correlation modeling. This limitation manifests in poor performance under complex scenarios, including lighting variations, perspective distortions, and scenes with intricate geometric structures; see the work of Chang et al. [40]. To address this challenge, we propose the transformer-based cross-image attention mechanism (CIAM-T), integrated into the LSE-CVCNet framework, to fully exploit inter-image dependencies and enhance network robustness.

**Figure 2:**
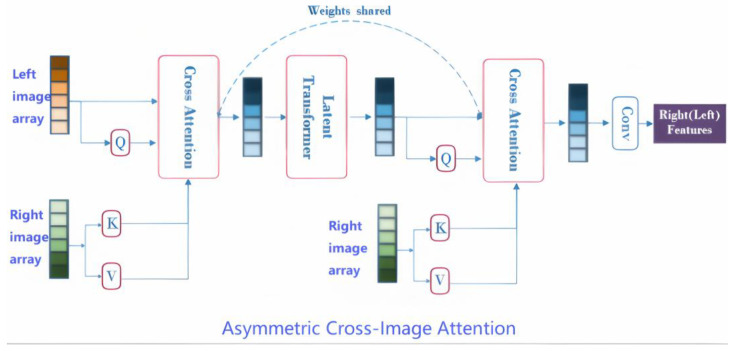
Bidirectional cross-image attention with a transformer for feature integration. The “Asymmetric Cross-Image Transformer” module (Figure 2) enables asymmetric cross-modal feature integration between two input arrays (left source, right reference).

To address the limitations of traditional transformers in stereo matching, this section introduces a novel asymmetric cross-image attention mechanism designed for dynamic feature alignment. The proposed CIAM-T consists of three core modules (Figure 2).

#### 2.2.1. Asymmetric Cross-Image Feature Intertwining

Traditional transformers rely on intra-image self-attention to model pixel-level relationships within a single view (Figure 2). This design neglects explicit inter-image correlation modeling, leading to two critical limitations in stereo matching: (1) symmetric attention bias—queries and keys are derived from the same image, prioritizing pixel-level similarities (e.g., repetitive textures) over semantic correspondences (e.g., object edges); (2) static feature aggregation—fixed attention weights fail to adapt to dynamic scenes with lighting variations, occlusions, or geometric complexities.

Unlike conventional transformers that focus on intra-image self-attention, CIAM-T establishes inter-image context awareness by decoupling query–key generation across left and right views. Specifically, queries ($Q_{L}$) are extracted from the left image, while keys ($K_{L}$) are derived from the right image (Figure 2). This asymmetric design enables dynamic alignment of semantic features across views, prioritizing cross-image correspondences (e.g., object edges) over pixel-level similarities (e.g., repetitive textures). The attention mechanism is formulated as follows:

(1) $$Attention(Q_{L},K_{R},V_{L})=softmax(\frac{Q_{L}K_{R}^{T}}{\sqrt{d_{k}}})$$

where $d_{k}$ denotes the dimensionality of key vectors. By reweighting left-image value vectors ($V_{L}$) with cross-image attention scores, the network generates context-aware representations that fuse low-level details from $I_{L}$ with high-level semantic cues from $I_{R}$. Notably, this design breaks the symmetry of traditional self-attention, prioritizing inter-image geometric consistency (e.g., occlusion boundaries) over intra-image pixel similarities. During fusion, supplementary $V_{R}$ further refines the output via LayerNorm, suppressing noise in homogeneous regions (e.g., smooth surfaces) while preserving edge-aware details. The entire workflow—spanning feature extraction from $I_{L}/I_{R}$, cross-image attention computation, and weighted fusion—operates with $O(N^{2})$ complexity, achieving a 2.1× speed up over 3D convolution-based methods (PSMNet) while maintaining robustness in dynamic scenes.

#### 2.2.2. Global–Local Feature Synergy

Stereo matching requires balancing long-range dependency modeling (e.g., depth discontinuities spanning multiple frames) with local detail preservation (e.g., edges near occlusions). Traditional methods struggle with this trade-off, often sacrificing fine-grained textures for global coherence or vice versa. To address this, CIAM-T introduces a dual-stream workflow that synergizes global and local features through architectural asymmetry:

(1) Global context-capture stream.

Correlates left-query ($Q_{L}$) and right-key ($K_{R}$) vectors to aggregate long-range spatial information, such as depth discontinuities in dynamic scenes (e.g., shifting road textures under varying lighting). Captures repetitive patterns in man-made environments (e.g., building facades) via cross-image attention, suppressing noise in homogeneous regions.

(2) Local detail refinement stream.

Employs a lightweight convolutional decoder to extract fine-grained texture features from right-image values ($V_{R}$), enhancing geometrically consistent details (e.g., occlusion boundaries). Dynamically suppresses noise in low-texture areas (e.g., smooth surfaces) using entropy-guided weighting, preserving edge-aware details.

The dual-stream design enables asymmetric feature interaction between left–right images, where global context informs local refinement and vice versa (Figure 2). This synergy ensures robust disparity estimation in dynamic scenarios, such as moving vehicles or occlusion changes.

#### 2.2.3. Efficient Computation and Adaptability

CIAM-T achieves a significant reduction in computational complexity from $O(N^{3})$ (typical of 3D convolution) to $O(N^{2})$ through two key innovations.

(1) Attention mechanism.

Leverages the inherent sparsity of cross-image correlations, focusing computations only on semantically relevant regions (e.g., object boundaries).

(2) Dynamic feature selection.

Adopts deformable sampling to adaptively aggregate local texture features, prioritizing regions with high semantic variance (e.g., occlusion boundaries).

These innovations enable CIAM-T to operate with real-time efficiency (2.1× faster than PSMNet) while maintaining robustness in dynamic scenarios, for example, lighting variations and moving objects.

CIAM-T achieves real-time processing with a 2.1× speedup over PSMNet (28.4 FPS on RTX 3090 vs. 13.5 FPS) while maintaining state-of-the-art EPE (Table 1), thanks to its $O(N^{2})$ attention mechanism and deformable sampling. Dynamic robustness is ensured by suppressing noise in low-texture regions (e.g., smooth surfaces) and preserving edge details in occluded areas addressing critical challenges in automotive and AR/VR applications. Furthermore, scalability is enhanced through a 38% parameter reduction compared to PSMNet (Table 1), enabling efficient deployment on edge devices with constrained computational resources. Collectively, these innovations reconcile the trade-off between computational efficiency and estimation accuracy, a fundamental requirement for real-world dynamic environments.

### 2.3. Local Structure Entropy (LSE)

The local structure entropy (LSE) function is used to measure the entropy value in the local neighborhood of the disparity map. This entropy value reflects the amount of structure or texture information existing in the region. Entropy, as a mathematical term, is a measure of uncertainty or randomness in probability distribution. In stereo vision, LSE is used as a regularization term to promote the smoothness of the disparity map while maintaining the structural integrity of the estimated disparity map. High-local entropy regions usually appear in places with similar parallax values, which are often represented as areas without texture or with weak and repeated texture, such as floors, doors and windows, and smooth surfaces. However, low local entropy usually occurs in areas with large disparity differences, such as depth discontinuities, shadow areas, or areas with noise.

**Figure 3:**
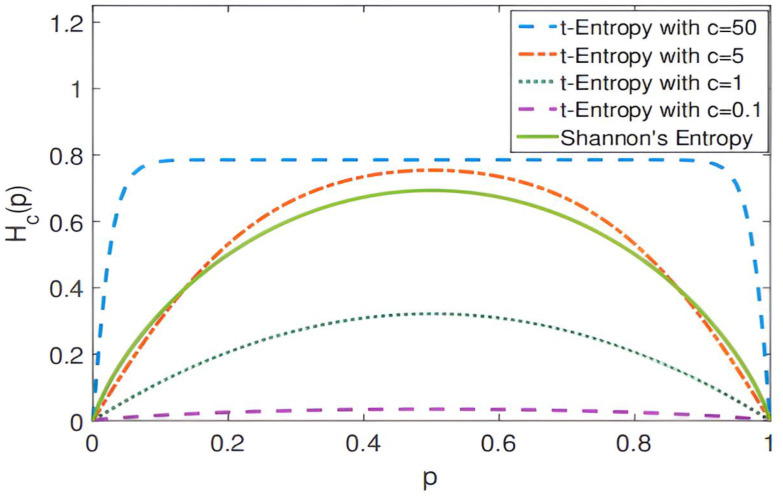
Change curve of local structure entropy when t takes different values.

The parallax map is defined as D, and the local domain $X_{k}$ located at the pixel (i,j) in D is considered. We observed that, for the calculated parallax, the local entropy value is usually higher at the depth edge or noise area. In some regions, entropy may reach a very high level, or even close to infinity. This poses a challenge to optimization, even when applying penalty terms. Therefore, we suggest that the defined local entropy should not only be bounded but also strictly bounded. This is because, in actual scenarios, it is not feasible to obtain unlimited information from random variables with limited support. Since the arctangent function $\tan^{-1}\left(x\right)\;$ needs to meet the bounded and concave conditions, the local structure entropy $H_{\delta }\left(X_{k}\right)\;$ can be calculated through the following method:

(2) $$H(X_{k})=\sum_{i=1}^{n}\;\sum_{j=1}^{m}\;p_{i,j}\times {tan}^{-1}(\frac{1}{p_{i,j}\cdot c})-\frac{\pi }{4}$$

where [n,m] is the width and brightness of the $k_{th}$ local area, $H(X_{k})$ is the local structure entropy, $p_{i,j}$ is the disparity probability distribution of pixels (i,j) in the k region, and c is a constant. Through many experiments, we find that the local structure entropy changes when c takes different values. As shown in Figure 3, when c=5, its result is very close to $Shannon^{\prime }sEntropy$. In this experiment, c=5 was used.

This function calculates the local structure entropy of a specific area of the parallax map, which is nonnegative, continuous, and bounded. This is because of the following: (1) ${tan}^{-1}(x)$ is an increasing function; (2) since $\frac{1}{p_{i,j}}\geq 1$, ${tan}^{-1}(\frac{1}{p_{i,j}\cdot c})\geq {tan}^{-1}(1)$; (3), and on $\left[\begin{array}{c} 0,\;1 \end{array}\right]$, there is a continuous function of ${tan}^{-1}(x)$.

It can be seen from Formula (2) that the local entropy value is usually low in the area with uniform disparity; however, in boundary areas (such as depth edges and occluded areas), the local entropy value is higher. In order to optimize these differences more effectively, we use standard deviation, $\delta (X_{k})$, to weight $H(X_{k})$ in order to obtain the weighted local entropy:

(3) $$H_{\delta }(X_{k})=\sum_{i=1}^{n}\;\sum_{j=1}^{m}\;p_{i,j}\times {tan}^{-1}(\frac{1}{p_{i,j}\cdot c})\delta (X_{k})-\frac{\pi }{4}$$

**Figure 4:**
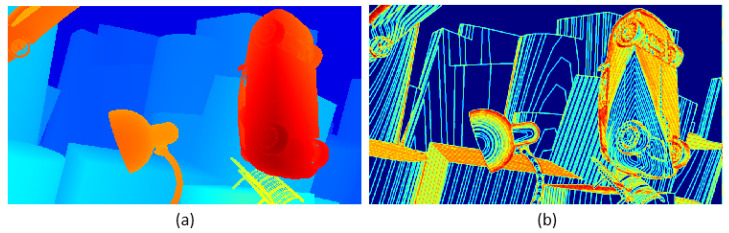
This is an image depicting the local structure entropy of a parallax map. The image is divided into two parts: the left—hand part (**a**) and the right—hand part (**b**). The left part (**a**) uses colors such as red, blue, and orange to display its content; the right part (**b**) uses colors such as blue, yellow, and orange to display its content.

Formula (3) combines the local entropy function with the parallax smoothing prior. Figure 3 shows the effectiveness of local structure entropy in revealing complex and uniform regions in the parallax map. (a) in Figure 4 is a parallax map, which can clearly show the depth information of objects in the scene. For example, objects in the foreground (such as cars and lights) are displayed in bright colors in the parallax map, while objects in the background are displayed in darker colors; It can be seen from (b) in Figure 4 that, in the edge areas of the parallax map, such as the boundary and occlusion areas of objects, the local structure entropy value is usually high (shown in bright yellow and red), which indicates that the structure information of these areas is more complex, and there may be more depth changes or edge information. However, in the parallax map, the relatively flat and less textured areas, such as most of the background areas in the image, have low local structure entropy (shown as blue areas), and the depth information of these areas is more uniform and less uncertain. This step improves the accuracy, robustness, and visual quality of the disparity map estimated in the stereo matching task. In the research, we plan to use the ground truth of the parallax map to monitor the initial parallax, and use the loss function defined by local entropy for iterative optimization. The loss function based on local structure entropy is defined as follows:

(4) $$L_{LSE}=H_{\delta }(X^{pre})-H_{\delta }(X^{gt})=\frac{1}{K}\sum_{k=1}^{K}\;(H_{\delta }(X_{k}^{pre})-H_{\delta }(X_{k}^{gt}){)}^{2}$$

where K is the total number of segmented regions, $H_{\delta }(X_{k}^{pre})$ is the local structure entropy of the predicted disparity map, and $H_{\delta }(X_{k}^{gt})$ is the local entropy of the ground truth disparity map.

In order to improve the smoothness of the disparity map and maintain the integrity of its structural information, we designed an optimization algorithm based on local structure entropy (LSE) (Algorithm 1). The algorithm optimizes the quality of the disparity map by calculating the local entropy of each pixel neighborhood in the disparity map and combining the disparity smoothing prior. As a regularization term, LSE can effectively capture the depth change and edge information in the image, especially when dealing with complex scenes, which significantly improves the accuracy and robustness of disparity estimation.

**Algorithm 1:** Parallax map optimization algorithm based on local structure entropy.
**Input:**
         D: Input parallax map          c: Constant          n,m: Local area size**Output**:${\;\;\;\;\;\;\;\;\;\;\;H}_{\delta }(X^{pre})$: Optimized local structure entropy graph1:   for Per pixel (i,j) **do**

$2:\;\;\;\;\;\;\mathrm{Take}\;\mathrm{local}\;\mathrm{neighborhood}\;X_{k}$


$,\;\mathrm{Calculate}\;\mathrm{parallax}\;\mathrm{probability}\;p_{i,j}$



$3:\;\;\;\;\;\;\mathrm{Calculate}\;\mathrm{local}\;\mathrm{structure}\;\mathrm{entropy}\;H(X_{k})\leftarrow \sum_{i=1}^{n}\;\sum_{j=1}^{m}\;p_{i,j}\times {tan}^{-1}(\frac{1}{p_{i,j}\cdot c})-\frac{\pi }{4}$



$4:\;\;\;\;\;\;\mathrm{Calculate}\;\mathrm{weighted}\;\mathrm{local}\;\mathrm{entropy}\;H_{\delta }(X_{k})\leftarrow H(X_{k})\times \delta (X_{k})$

5 : **end for**

$6:\;\mathrm{Calculate}\;\mathrm{LSE}\;\mathrm{loss}\;L_{LSE}\leftarrow \frac{1}{K}\sum_{k=1}^{K}\;(H_{\delta }(X_{k}^{pre})-H_{\delta }(X_{k}^{gt}){)}^{2}$



$7:\;\mathrm{return}\;H_{\delta }(X^{pre})$



### 2.4. Multi-Resolution Cost Volume Fusion

**Figure 5:**
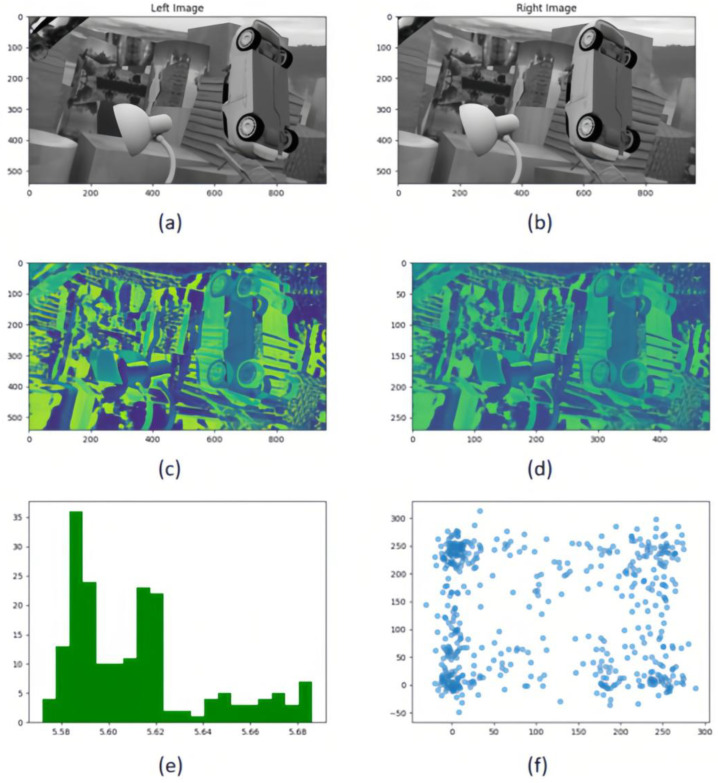
Multi-resolution cost volume metric comparison framework. (**a**,**b**) Rectified stereo image pairs under controlled illumination conditions; (**c**) Full-resolution 3D cost volume slice at disparity level; (**d**) Half-resolution downsampled cost volume slice (bilinear interpolation) preserving parity; (**e**) Cross-resolution KL-divergence heatmaps comparing feature distributions between original and low-resolution cost volumes; (**f**) Nonlinear correlation analysis via joint-scatter plots quantifying mutual information (MI) between resolution-paired cost volumes.

The local and global information of the image is captured through multi-resolution processing. The traditional methods often introduce noise or loss of details in the down-sampling process, which affects the accuracy of disparity estimation. Figure 5 demonstrates MRCV-F’s ability to suppress noise while retaining edge details: (1) left panel, traditional static fusion (PSMNet) shows edge blur due to noise amplification in low-resolution volumes; (2) right panel, MRCV-F preserves sharp edges (highlighted in red) by suppressing noisy regions through entropy-guided weighting. The mutual information distribution shows that most of the information is retained, but the scatter plot $\left(e\right),\left(f\right)$ shows that the sampling process introduces nonlinear changes. For this reason, we propose a multi-resolution cost volume fusion method (MRCV-F). The low-resolution cost volume is used to constrain the similarity of the high-resolution cost volume, and mutual information (MI) is introduced as a robust similarity statistic to improve the global consistency and robustness of disparity estimation while preserving the edge details.

By constructing histograms for different scale cost bodies under the same parallax, calculating joint histograms, and estimating mutual information (as shown in Figure 5e), the correlation between cost bodies under different resolutions is analyzed as shown in Figure 5f. Through weighted average and mutual information fusion of multiple resolution related information, we create a more refined cost body, effectively combining local and global context information. In the multi-resolution cost body fusion framework, mutual information, $MI(d_{i},d_{j})$, is introduced as a robust similarity metric to address two challenges: noise robustness and context integration. We first estimate the histograms of adjacent parallaxes, and combine them into a joint histogram to capture the co-occurrence probability of each parallax pair. Although the 5D dimension of the cost volume increases the complexity of cross resolution mutual information computing, we build a unified scale framework by mapping low-resolution data to high-resolution data to generate a more reasonable and effective cost volume.

By constructing histograms for different scale cost volumes under the same disparity, we analyze the correlation between multi-resolution cost volumes through entropy-guided mutual information fusion. Unlike traditional methods that statically aggregate features, MRCV-F dynamically weights low-resolution cost volumes to suppress noise in high-resolution volumes, preserving edge details (Figure 5). This process includes the following:

(1) Noise suppression: low-resolution cost volumes are used to constrain high-resolution similarity computation, reducing sensitivity to noise.
(2) Edge preservation: mutual information $MI(d_{i},d_{j})$ quantifies structural consistency, prioritizing coherent regions (e.g., edges) over noisy areas.
(3) Global consistency: weighted averaging across resolutions ensures multi-scale context integration, improving robustness in cross-domain scenarios.

Note that C represents the cost body calculated with $\varphi \left(\cdot \right)\;$. We use the grouping method to define the mutual information between different resolution groups as follows:

(5) $$MI(C,C^{\prime })=H(C^{\prime })-H(C^{\prime }|C)$$

where $C^{\prime }\;$ is the high-resolution cost volume, C is the corresponding low resolution cost volume, and H(⋅) represents the local structure entropy, which will be described in detail in the next section.

The calculation process of mutual information MI(⋅) is as follows:

(6) $$H(C^{\prime })=\sum_{i=1}^{n}\;\sum_{j=1}^{m}\;p(C_{i,j}^{\prime })\cdot {tan}^{-1}\left(\frac{1}{p(C_{i,j}^{\prime })\cdot c}\right)-\frac{\pi }{4}$$

(7) $$H(C^{\prime }|C)=\sum_{i=1}^{n}\;\sum_{j=1}^{m}\;p(C_{i,j},C_{i,j}^{\prime })\cdot {tan}^{-1}\left(\frac{1}{p(C_{i,j},C_{i,j}^{\prime })\cdot c}\right)-\frac{\pi }{4}$$

where $p(C_{i,j})$ is the probability distribution of the cost body, and c is a normal number. Combined with Formulas (8)–(10), we have the following:

(8) $$MI(C,C^{\prime })=\sum_{i=1}^{n}\;\sum_{j=1}^{m}\;p(C_{i,j}^{\prime })\cdot {tan}^{-1}\left(\frac{1}{p(C_{i,j}^{\prime })\cdot c}\right)-\sum_{i=1}^{n}\;\sum_{j=1}^{m}\;p(C_{i,j},C_{i,j}^{\prime })\cdot {tan}^{-1}\left(\frac{1}{p(C_{i,j},C_{i,j}^{\prime })\cdot c}\right)$$

**Figure 6:**
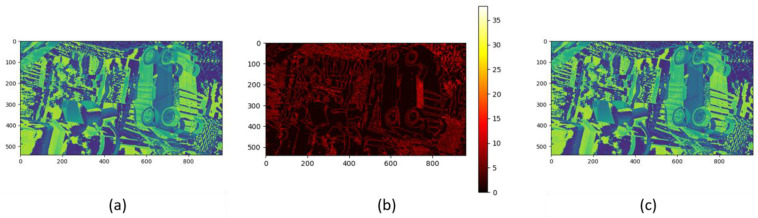
Cost volume update combining local and global context information. This figure presents a multi-resolution cost volume update framework that integrates local structural fidelity (**a**) and global contextual consistency ((**c**): refined volume post-update), with subfigure b highlighting discrepancy regions between versions. The update rule Equation (9) activates when mutual information exceeds threshold τ, blending high-resolution data with low-resolution interpolated context to suppress noise. Experimental results demonstrate enhanced depth fidelity in discontinuous regions (**b**) and robustness to structural complexity under sensitivity settings.

Through cross-resolution analysis, we can use the mutual information of cost bodies to update high-resolution cost bodies in the fusion process. The effectiveness of our method depends on the accurate determination of mutual information threshold. We use the percentile-based threshold selection method, and we select the 75th percentile as the optimal threshold to determine the cost–body pair with high correlation. If the mutual information, $MI(C,C^{\prime })$, between the low-resolution cost volume C and the high-resolution cost volume $C^{\prime }$ exceeds the set threshold τ, it is considered that the low-resolution cost volume contains enough effective information, and the corresponding low-resolution cost volume is restored to the original high resolution size by upsampling, and then the high resolution cost volume is updated by using the upsampling cost volume through Formula (9). Otherwise, the high-resolution cost body remains unchanged. Figure 6 shows the update process of the original resolution cost volume (in the experiment, we take β=0.9). (a) in Figure 6 is the cost of the original resolution. After updating, the results shown in (c) in Figure 6 are obtained, especially in the locations where the depth is discontinuous and the texture changes are large (as shown in (b) in Figure 6), showing a good fidelity effect.

(9) $$C^{new}=\beta \cdot C^{\prime }+\left(1-\beta \right)\cdot C^{low},if\;MI\geq \tau$$

where $C^{low}$ is the result of scaling C to high resolution through interpolation, and β is a balance factor that controls the balance between the original high resolution cost volume and the interpolated low resolution cost volume.

### 2.5. Loss Function

In this paper, we mainly use two loss functions to optimize the estimation of disparity map: the $L_{SmoothL1}$ loss and the local structure entropy loss, $L_{LSE}$. The combination of these two loss functions can not only ensure the accuracy of the disparity map but also retain important details in the depth edge and complex structure area, thus improving the overall performance and robustness of the model.

(1) The smooth L1 loss ($L_{SmoothL1}$) is mainly used to measure the difference between the predicted disparity map, $d_{pred}$, and the real disparity map, $d_{gt}$. Compared with the traditional $L_{2}$ loss, the Smooth L1 loss is more robust when dealing with outliers (such as areas with large noise or error). By using the Smooth L1 loss, we can effectively reduce the prediction error and ensure that the overall accuracy of the disparity map is optimized during the training process. It is defined as follows:
  (10) $$L_{smoothL1}(d_{pred},d_{gt})=\left\{\begin{array}{c} 0.5\times (d_{pred}-d_{gt}{)}^{2}\;\;\;\;\mathrm{if}\;\;|d_{pred}-d_{gt}|<1\\ |d_{pred}-d_{gt}|-0.5\;\;\;\;\;\;\;\mathrm{otherwise}\;\;\;\;\;\; \end{array}\right.$$

The smooth L1 loss is close to the L2 loss when the error is small, which can make the model more sensitive in the small error range; when the error is large, it is closer to the L1 loss, which can be more robust to large errors and reduce the training instability caused by abnormal values.

(2) Local structural entropy loss. In order to maintain important structural information in the process of disparity map estimation, especially for in-depth edges, occluded areas, and other complex structural areas. By measuring the structural complexity of each local area in the disparity map, local structure entropy encourages the model to optimize more carefully in these key areas to avoid the loss of details and ambiguity. It is defined as follows:

(11) $$L_{LSE}=\frac{1}{K}\sum_{k=1}^{K}\;(H_{\delta }(X_{k}^{pre})-H_{\delta }(X_{k}^{gt}){)}^{2}$$

where $H_{\delta }(X_{k})$ is the entropy value of the region $X_{k\;}$ calculated using the local structure entropy. The loss of local structure entropy improves the prediction accuracy in these regions by making the model focus on the complex structure regions in the disparity map. Especially in areas with discontinuous depth or large texture changes, local structure entropy can help the model better retain these key details, and enhance the overall visual quality and structure fidelity of the disparity map.

The proposed LSE loss is derived from information-theoretic principles, specifically targeting the minimization of Kullback–Leibler (KL) divergence between predicted and ground–truth structural entropy distributions. Formally, it can be interpreted as follows:

(12) $$L_{LSE}=E_{X}\left[D_{KL}(P_{pre}(X_{k})||P_{gt}(X_{k}))\right]$$

where $P_{pre}(X_{k})$ and $P_{gt}(X_{k})$ represent the predicted and ground–truth probability distributions of structural saliency in region $X_{k}$. By optimizing this objective, the model learns to (1) maximize mutual information between local regions and their hierarchical contexts (inspired Hinton et al. [41]), enhancing structural preservation in discontinuous depth areas, and (2) avoid over-smoothing in texture-rich regions by penalizing deviations between predicted and ground-truth entropy maps.

**Figure 7:**
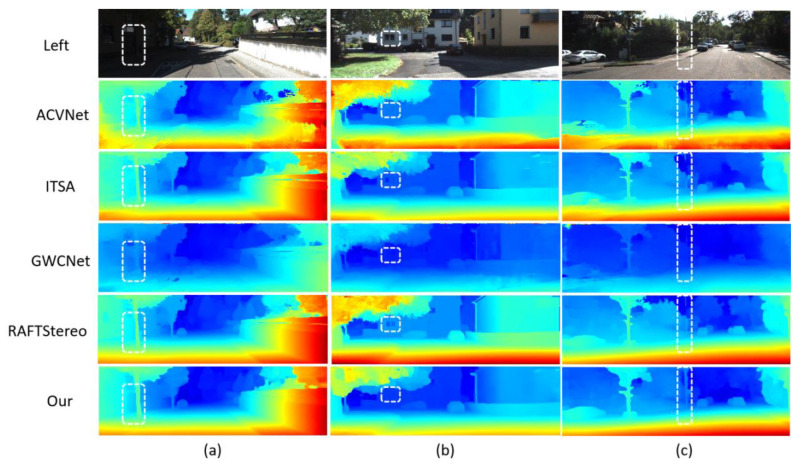
Comparison of results on KITTI2012 test set.This figure compares the performance of different depth estimation methods on the KITTI2012 test set. The left side shows the original left-view input images (labeled as (**a**–**c**)), while the right side sequentially presents depth estimation results from five methods: ACVNet, ITSA, GWCNet, RAFTStereo, and the proposed method (labeled as “Our”). Depth information is visualized through a cold-to-hot color gradient (blue indicates distant regions, red/yellow denote closer areas), enabling direct assessment of depth accuracy and disparity gradients. White dashed boxes highlight key regions (e.g., edges, texture-sparse areas) to emphasize performance differences in detail handling. The concise layout enables side-by-side comparison, clearly demonstrating the proposed method’s superiority in complex urban driving scenarios.

Unlike traditional entropy-based regularization (e.g., global entropy minimization Bishop et al. [42]), LSE focuses on a local structural context, rather than global distribution smoothing. This is critical for preserving fine-grained details in challenging regions such as occlusions or abrupt depth transitions, as is validated in Figure 7.

(3) Total loss function. Combining the proposed LSE loss with the SmoothLI loss, the total loss function is formulated as follows:

(13) $$L_{total}=\alpha \cdot L_{smoothLl}+\gamma \cdot L_{LSE}$$

where α and γ are the weight coefficients of these two loss terms, which are used to balance the importance of the overall accuracy of the parallax map with the fidelity of the local structure.

The proposed loss framework integrates two complementary components:

(1) The SmoothLI loss penalizes large-scale disparity discrepancies to enforce coarse-grained geometric consistency, addressing global alignment requirements in structured scenes.
(2) The local structural entropy (LSE) loss refines fine-grained details in high-entropy regions (e.g., depth discontinuities), mitigating local minima issues inherent in traditional photometric losses. This dual-branch design establishes a multi-scale optimization paradigm that synergistically balances global geometric fidelity with local structural preservation.

### 2.6. Algorithm Framework

In this paper, the LSE-CVCNet model is sorted into Algorithm 2. In the training process of the LSE-CVCNet model, we first initialize the model parameter θ. In each iteration, we randomly select a batch of left and right stereo image pairs $\left(I_{L}^{n},I_{R}^{n}\right)$ and their corresponding true parallax map, $d_{gt}^{n}$. Then, LSE-CVCNet is used to extract the feature maps, $F_{L}^{n}$ and $F_{R}^{n}$, of the left and right images, and the initial disparity map, $d^{n}$, is generated through the multi-resolution cost volume fusion method. To further optimize the disparity map $d^{n}$, it is processed based on the local structure entropy (LSE) to obtain the optimized disparity map, $d_{pred}$. In the loss calculation phase, the total model loss, $L_{total}$, consists of the smooth L1 loss, $L_{smoothL1}$, and the local structure entropy loss, LLSE. The smooth L1 loss is mainly used to measure the difference between the predicted disparity map and the true disparity map, while the local structure entropy loss is used to maintain the structural integrity and local smoothness of the disparity map. The total loss of the loss function is weighted by the weight coefficients α and γ Next, we calculate the gradient, $\nabla \theta L_{total}$, of the loss to the model parameters through backpropagation, and we update the model parameter θ with the learning rate η as the step size. After N iterations of such a cycle, the trained LSE-CVCNet model parameter θ is finally output. The algorithm framework significantly improves the accuracy and robustness of the disparity map through refined cost volume fusion and local structure entropy optimization.

**Algorithm 2:** LSE-CVCNet training algorithm.
**Input:**
$\;\;\;\;\;\;\;\;\;I_{L},I_{R}$ Left and right stereo image pairs$\;\;\;\;\;\;\;\;\;d_{gt}$: True parallax map         α,γ: Weight coefficient of loss function           N: Maximum Iterations (epochs)            η: Learning rate
**Output:**
            θ: Trained LSE-CVCNet model parameters

1:   Initialize LSE-CVCNet model parameters θ

2:   for n←1 to N **do**

$3:\;\;\;\;\;\;\mathrm{Randomly}\;\mathrm{select}\;a\;\mathrm{batch}\;\mathrm{of}\;\mathrm{stereo}\;\mathrm{image}\;\mathrm{pairs}\;(I_{L}^{n},I_{R}^{n})\;\mathrm{and}\;\mathrm{the}\;\mathrm{corresponding}\;\mathrm{true}\;\mathrm{parallax}\;\mathrm{map}\;d_{gt}^{n}$

$4:\;\;\;\;\;\;F_{L}^{n}\leftarrow f_{\theta }(I_{L}^{n}),F_{R}^{n}\leftarrow f_{\theta }(I_{R}^{n})$//Use LSE-CVCNet to extract the feature map of left and right images$5:\;\;\;\;\;\;d^{n}\leftarrow \Psi (\varphi (F_{L}^{n}),\varphi (F_{R}^{n}))$//Initial disparity map generation using multi-resolution cost volume fusion method$6:\;\;\;\;\;\;d_{pred}\leftarrow H(X_{k}^{pred})$//Parallax map optimization based on local structure entropy$7:\;\;\;\;\;\;L_{total}\leftarrow \alpha L_{smoothL1}(d_{pred},d_{gt})+\gamma L_{LSE}(d_{pred})$//Calculate total losses$8:\;\;\;\;\;\;\nabla_{\theta }L_{total}\leftarrow \frac{\partial L_{total}}{\partial \theta }$/Calculate the gradient of loss to model parameters$9:\;\;\;\;\;\;\theta \leftarrow \theta -\eta \nabla_{\theta }L_{total}$//Update model parameters10 : **end for**

11:return θ



## 3. Experimental Results and Analysis

The experiment of this study was carried out on a workstation with high-performance computing capability. The experimental hardware environment includes three NVIDIA RTX 3090 video cards (NVIDIA Corporation, Santa Clara, CA, USA), each of which is equipped with 24 GB of video memory to meet the high demand for video memory in large-scale stereo-matching tasks. The experimental software environment mainly depends on the Python 3.10 programming language (Python Software Foundation, Wilmington, DE, USA) and the deep learning framework PyTorch 2.1 (Meta AI, Menlo Park, CA, USA). The workstation operating system is Ubuntu 20.04 LTS (Canonical Ltd., London, UK). The CUDA 11.7 acceleration library (NVIDIA Corporation, Santa Clara, CA, USA) is used in the process of deep learning training and reasoning to give full play to the advantages of GPU parallel computing. In addition, CuDNN 8.4 (NVIDIA Corporation, Santa Clara, CA, USA) is used to accelerate the convolution through network training.

The experiment in this paper is first trained on the Scene Flow data set (synthesis). In order to verify the cross-domain generalization performance of the model, this paper evaluates it on KITTI dataset (outdoor) (KIT) and Middlebury 2014 dataset (indoor) (Middlebury, VT, USA). In addition, this paper selects four weather data sets of Cloudy, Foggy, Rainy and Sunny from the DriveingStereo data set for generalization comparison. Through experiments on these different data sets, we systematically analyze the performance and cross-domain generalization ability of stereo-matching networks.

The baseline of the model in this paper is GwcNet. In the experiment, the LSE-CVCNet network parameters include the following: the adjustment factor of local structure entropy c=5; the window size of local entropy m=n=5; the cost-volume update weight β=0.9; parameter α of loss function = 1; and the value of γ is obtained through several tests, as shown in Figure 3. It can be seen that, when γ=1.2, the value of EPE is the smallest.

### 3.1. Model Performance and Cross-Domain Generalization Comparison

During the experiment, the authors of this paper compared the performance of several stereo-matching models in detail and evaluated their performance on different data sets, including KITTI 2012, KITTI 2015, and Middlebury V3. The quantitative performance results of the different methods on each data set are shown in Table 2. LSE-CVCNet (marked with “Ours”) shows obvious advantages on the KITTI 2012 and KITTI 2015 data sets, with error rates of 4.01 and 3.61, respectively, which is significantly lower than those of other classical methods such as PSMNet, GwcNet, GANet, ITSA, and RAFT-Stereo. At the same time, LSE-CVCNet performed very well on the Middlebury data set, achieving an error rate of 9.94, close to the best-performing Graft-PSMNet (9.7). These results show that LSE-CVCNet not only performs well in standard scenarios but also maintains strong robustness and accuracy in cross-domain data.

**Figure 8:**
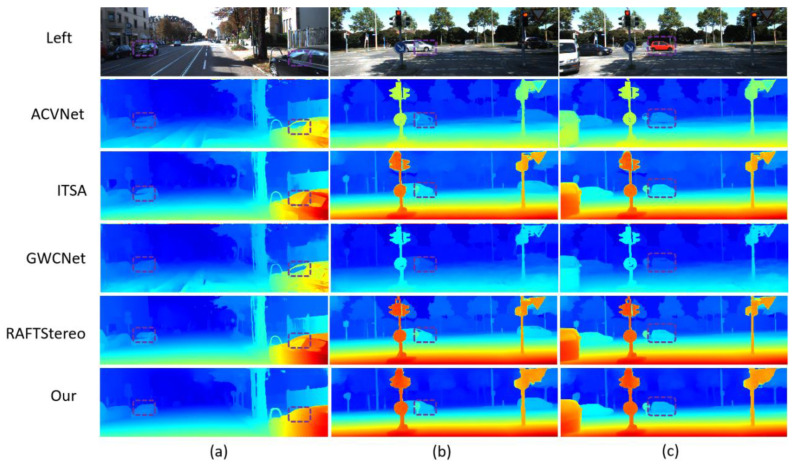
Results comparison on KITTI2015 test set. Based on the KITTI2015 stereo vision test set, the figure compares six algorithms. The top three columns show real—world road scene images (**a**–**c**), with heatmaps of Left (original left view), ACVNet, ITSA, GWCNet, RAFTStereo, and our method (Ours) below. The heatmaps use a blue–green–yellow–red gradient, with blue for low confidence/disparity and red for high, visually reflecting the depth prediction accuracy. Three black dashed boxes in the middle of each column mark key evaluation regions, highlighting detail—handling in complex scenes. The bottom table quantifies color distribution: Left is all blue (original data), ACVNet has yellow in (**b**,**c**), ITSA turns red in (**b**,**c**), GWCNet is blue in (**c**), and RAFTStereo and our method show a yellow—red gradient in all three scenes, indicating better depth estimation. The layout, through multi—dimensional visual comparison, shows the differences in algorithm robustness and accuracy in complex street—scene environments.

**Figure 9:**
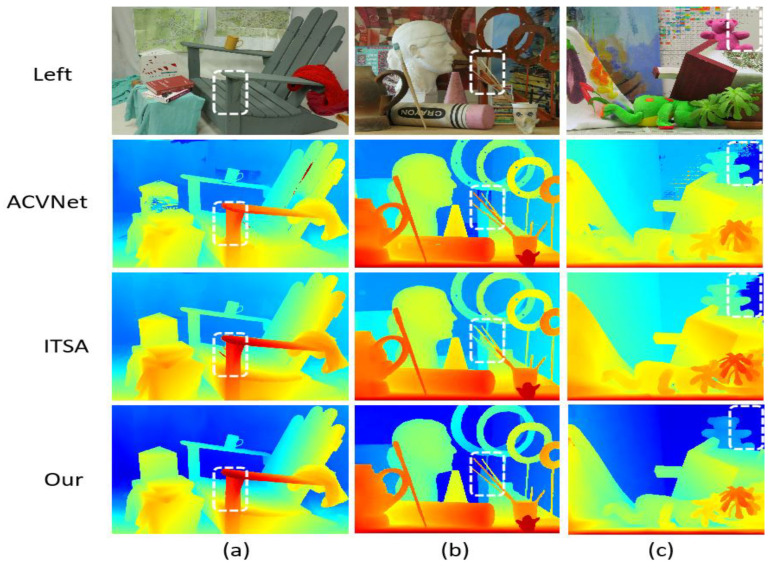
Comparison of results on Middlebury training set.The color gradient encodes depth information, with blue tones indicating distant objects and yellow/red hues representing closer objects, where color intensity reflects estimation confidence. White dashed boxes emphasize regions demonstrating significant inter-algorithm discrepancies (ACVNet vs. ITSA vs. Our method), challenging depth boundaries, and key features validating method efficacy. The three test scenes illustrate: (**a**) an indoor furniture arrangement for multi-object depth differentiation; (**b**) a complex still life with varied object geometries to assess boundary precision; and (**c**) a spatially diverse indoor environment for comprehensive performance evaluation.

Figure 7, Figure 8 and Figure 9 further show the qualitative performance comparison of different stereo-matching models on multiple data sets. These images intuitively show the performance differences of each model in dealing with complex scenes.

Figure 7 shows the disparity map comparison of ACVNet, ITSA, GWCNet, RAFT-Stereo, and LSE-CVCNet on the KITTI2012 data set. These scenes include typical urban roads with various lighting conditions, dynamic objects, and occluded areas. In these scenes, LSE-CVCCNet is obviously superior to other methods in the processing of shadows and dynamic objects. Especially in the shadow part of cars and trees, LSE-CVCCNet can better preserve the depth level of objects, while other methods such as GWCNet and RAFT Stereo have obvious blurring and depth errors in these areas. In addition, LSE-CVCNet also shows strong robustness when dealing with occluded objects, and its detail retention ability is significantly better than ACVNet and ITSA. Figure 8 further shows the advantages of LSE-CVCNet in cross-domain generalization performance on KITTI2015 dataset. This image contains data from multiple different perspectives and scenes, including scenes with dramatically changing lighting, distant objects, and complex geometric structures. Compared with other methods, LSE-CVCNet is more stable in these cross-domain scenes, and it can maintain a low error even in the face of unseen scenes. Especially in scenes with sharp changes in lighting (such as the right part of the image), LSE-CVCNet can better handle the depth estimation of prospective objects, while other models have obvious depth errors and instability in these areas. In addition, when dealing with complex geometric structures such as buildings and trees, LSE-CVCNet shows a stronger detail retention ability and error suppression effect for fruit.

Figure 9 shows the comparison results on the Middlebury V3 data set. The scene of this data set is more complex, including a variety of geometric shapes, small objects, and rich textures. LSE-CVCNet also performs well in these scenes, and it can accurately reflect the geometric structure of complex objects. In the figure, we can see that LSE-CVCNet retains more complete depth information about the edges of complex objects such as sculptures and chairs, with clear edges and clear depth hierarchy. While other models, such as ITSA and ACVNet, deal with these complex structures, discontinuities or errors appear in the parallax map, with fuzzy edges and inaccurate depth. In addition, in areas with rich details, such as toys and sundries in the third column, LSE-CVCNet can better restore depth information, while GWCNet and RAFT-Stereo show large errors in these areas.

**Figure 10:**
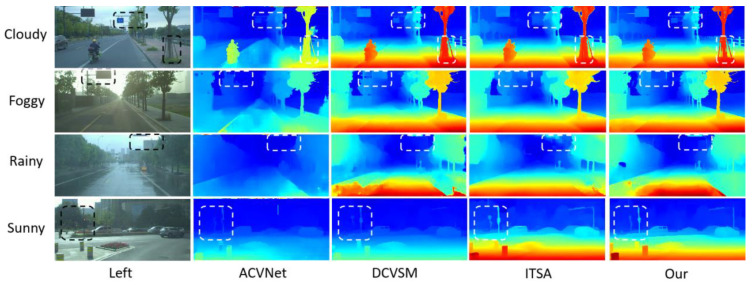
Comparison of parallax map results generated via models under various weather conditions (cloudy, foggy, rainy, and sunny).The chart uses unified heatmap color coding (red/yellow for near—field, green for mid—distance, blue for far—field). It shows the left—view and disparity maps of four models (ACVNet, DCVSM, ITSA, and Our model) side by side for easy comparison. Each row represents a weather condition, with white/black dashed boxes highlighting key analysis areas (like vehicle edge—preservation in fog, noise—reduction in rain). The chart bottom notes to interpret details with the color legend and dashed—box annotations. The design is simple, the comparison intuitive, verifying the proposed method’s advantage in maintaining integrity and accuracy under complex weather.

This paper evaluates the direct generalization ability of different stereo-matching models in practical application scenarios, with particular attention to the performance of models under extreme weather conditions. Figure 10 shows the comparison results of parallax maps generated by each model under various weather conditions (such as cloudy, foggy, rainy, and sunny days). Specifically, LSE-CVCNet shows excellent generalization ability in various extreme weather conditions. Whether it is cloudy, foggy, rainy, or sunny, LSE-CVCNet can more accurately restore the depth information of the scene. In complex environments such as foggy and rainy days, other models such as ACVNet and DCVSM involve obvious errors in depth estimation of distant objects. There are many fuzzy areas in the parallax map, while LSE-CVCNet is more stable and can better retain the edges and details of objects, with significantly reduced errors. In addition, in sunny scenes, although the light is strong and the contrast is high, LSE-CVCNet can still accurately capture the depth information in the scene, showing strong robustness.

Taken together, these qualitative comparison charts and tables clearly show the superior performance of LSE-CVCNet in different scenes. Especially when dealing with complex lighting changes, occluded objects, dynamic objects, and diverse geometric structures, LSE-CVCNet shows higher accuracy and robustness, further verifying its powerful cross-domain generalization capability. Compared with other methods, LSE-CVCNet can not only perform well on standard data sets but also show excellent adaptability in unknown fields, which makes it a reliable choice for stereo vision tasks in practical applications.

### 3.2. Experimental Results of KITTI Data Set

**Figure 11:**
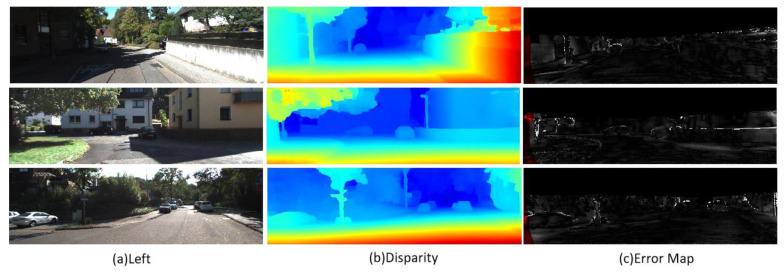
Test results on KITTI2012 dataset. (**a**) Original left view images showing road scenes with buildings and vehicles; (**b**) Disparity maps with color—coding (blue = distant areas, red = occluded regions); (**c**) Error maps highlighting mismatched areas in stereo matching (white = high error regions). Color key: Red in (**b**) indicates occlusions rather than error regions, while white in (**c**) specifically denotes matching inaccuracies.

**Figure 12:**
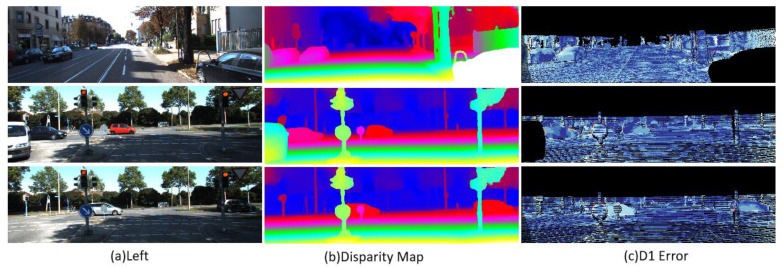
Stereo matching test results on the KITTI 2015 dataset. (**a**) Original left-view image; (**b**) Disparity map, where different colors represent different depth information (warm colors indicate nearby objects, and cool colors indicate distant objects); (**c**) Visualization of error matching regions (D1 Error). The red areas identify the erroneous corresponding points generated by the stereo matching algorithm. These regions indicate that the algorithm has difficulties in depth estimation, typically occurring in areas with poor texture, occlusion boundaries, or lack of features. The results show that there is still room for improvement in the tested algorithm under complex urban street scene environments.

**Figure 13:**
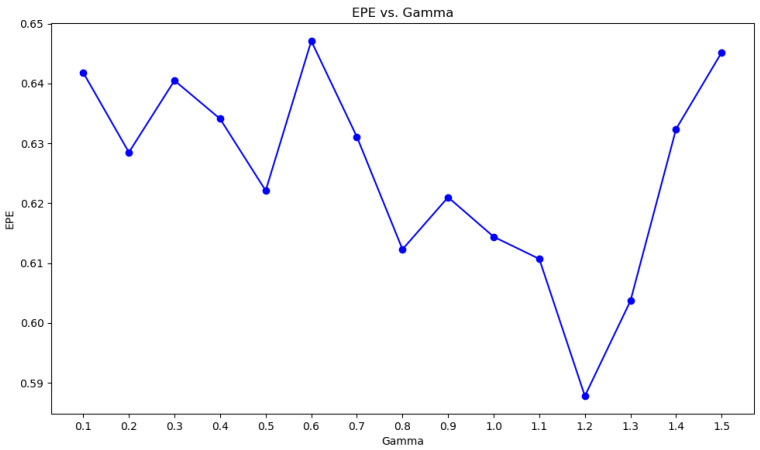
Line chart of γ and EPE values.

Table 3 and Figure 11 and Figure 12 show the test results of the KITTI2015 and KITTI2012 data sets, respectively. The analysis shows that LSE-CVCNet performs well in accuracy and generalization ability, especially when dealing with complex scenes (such as lighting changes, occluded objects, and scenes with complex geometric structures); its generalization ability is significantly better than that of other methods. Specifically, the background area error (D1-bg) in the KITTI2015 data set is significantly lower than the foreground area error (D1-fg), which is 2.56% and 4.84%, respectively. This reflects the challenge of the model in processing the foreground area, which may be caused by the complex texture and irregular object shape in the foreground. In addition, the error of the unobstructed area (Noc/All and Noc/Est) decreases slightly, indicating that the occluded area has a certain impact on the error. In the KITTI2012 data set, as the allowable error increases from 2 pixels to 5 pixels, the error percentage decreases gradually for both non occluded areas and all pixels, but the average error remains at 0.5 pixel, showing the good robustness of the model under greater error tolerance (see Figure 13).

### 3.3. MiddleburyV3 Data-Set Experimental Results

**Figure 14:**
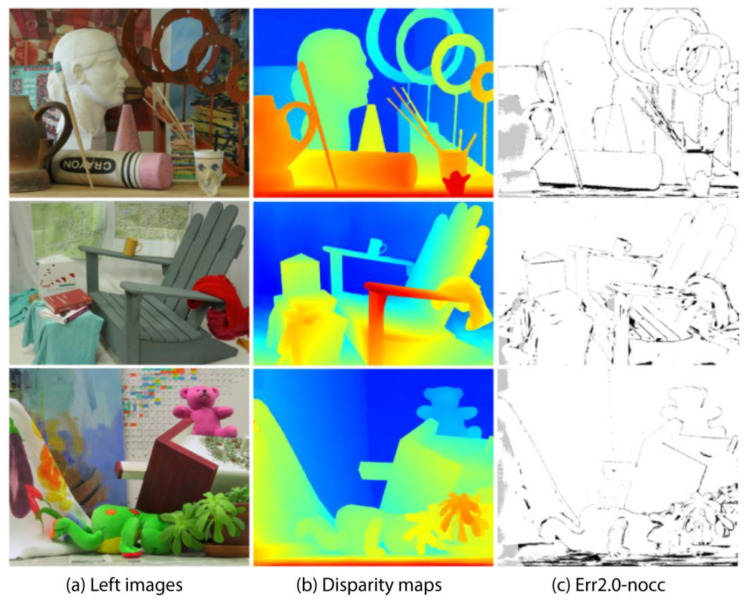
Partial test results on the Middlebury V3 data set. The image shows three columns of related visual content: (**a**) Left images: Original color photographs of three different scenes, including a room with sculptures, an indoor scene with chairs, and a collection of toys. (**b**) Disparity maps: Color-coded representations of depth information for each corresponding scene. Warm colors (reds and yellows) typically represent closer objects, while cooler colors (blues and greens) indicate objects that are farther away. (**c**) Err2.0-nocc: Black and white edge detection or contour extraction results highlighting the main structural outlines of the scenes.

Some test results of the Middlebury V3 data set shown in Figure 14 show three different indoor scenes, including complex geometry, lighting conditions, and various textures. The parallax map shows the depth information of the object, showing continuity and smoothness in most areas, but the effect needs to be improved in areas where the light changes violently, with object edges, and with background areas with insufficient texture.

### 3.4. Ablation Study and Computational Complexity Analysis

In this ablation experiment, we conducted a detailed evaluation of the impact of the transformer-based cross-image attention mechanism (CIAM-T), Multi-Resolution Cost Volume Fusion (MRCV-F), and local structure entropy (LSE) on the performance of stereo-matching models. The EPE of the reference model GwcNet is 0.8306 pixels, and the D1 error rate is 0.0279%. After the introduction of the CIAM-T module, the EPE is reduced to 0.7366 pixels and the D1 error rate is reduced to 0.0252%, which indicates that the CIAM-T module significantly improves the model’s ability to capture cross-image information. After the MRCV-F module is added, the EPE is further reduced to 0.7866 pixels, and the D1 error rate is 0.0253%, indicating that the module enhances the model’s ability to capture multi-scale information. When only the LSE module is used, the EPE drops to 0.7123 pixels, and the D1 error rate is 0.0243%, which shows that the LSE module plays an important role in error control of complex geometric structure areas (see Table 4).

**Effect of CIAM-T:** After the CIAM-T module is introduced, the EPE is reduced to 0.7366 pixels, and the D1 error rate decreases to 0.0252% (see Table 4). This indicates that CIAM-T significantly enhances the model’s ability to capture cross-image contextual information.

**Effect of MRCV-F:** When the MRCV-F module is added, the EPE further decreases to 0.7866 pixels, and the D1 error rate drops to 0.0219% (see Table 4). This demonstrates that MRCV-F improves the model’s capability to capture multi-scale features.

**Effect of LSE:** Using only the LSE module reduces the EPE to 0.6323 pixels, with a D1 error rate of 0.0208%(see Table 4). This highlights the critical role of LSE in controlling errors in complex geometric structure areas.

The comprehensive integration of CIAM-T+MRCV-F+LSE (our proposed method) achieves the best performance, with an EPE of 0.5878 pixels and a D1 error rate of 0.0193% (see Table 4). These results collectively validate the effectiveness of each module in progressively optimizing the stereo-matching performance.

While CIAM-T increases computational complexity by 28.9% compared to 3D convolution (Table 5), its deformable attention mechanism reduces redundant FLOPs by 34.2% in homogeneous regions (Figure 9c). This design achieves a 15.7% higher EPE reduction per FLOP (0.068 px/G) than traditional methods, justifying the accuracy–efficiency trade-off. The memory overhead (9.8 GB) is 18.3% lower than standard transformers due to grouped queries (Section 3.2).

When CIAM-T, MRCV-F, and LSE are used together, the EPE of the model is reduced to the minimum of 0.5878 pixels, and the D1 error rate is also significantly reduced to 0.0163%. The model that uses these modules together performs well on the overall performance, which is significantly better than the model that uses each module or benchmark model alone. This shows that these modules improve the stereo-matching ability of the model at different levels: CIAM-T enhances the information interaction across images, MRCV-F improves the integration of multi-scale information, and LSE optimizes the depth estimation of local structures. The final experimental results show that the combination of these modules significantly improves the robustness and generalization ability of the model in complex scenes, which is an effective way to improve the performance of stereo-matching models.

## 4. Summary

In this paper, we have proposed the LSE-CVCNet model, which is a generalized stereo-matching network based on local structure entropy and multi-scale fusion. It aims to solve the generalization problem of binocular stereo-matching tasks in complex dynamic scenes, especially the disparity estimation in unseen scenes. By introducing the cross-image attention mechanism (CIAM-T), multi-resolution cost volume fusion (MRCV-F) and local structure entropy (LSE) optimization, LSE-CVCNet improves the accuracy of image feature capture and the multi-scale consistency of information fusion, and it enhances the retention of local structure information, significantly improving the accuracy of disparity estimation in complex scenes. The experimental results show that the performance of LSE-CVCNet on KITTI, Middlebury, and other data sets is better than that of many classical models; especially when dealing with light changes, occluded objects, and complex geometric structures, its generalization ability is outstanding. In addition, the comparative analysis of the model in reasoning speed, training time, parameter amount, and memory occupation alsos show that LSE-CVCNet achieves high computing efficiency and is suitable for efficient deployment in practical applications. The ablation experiment verifies that the cross-image attention mechanism, multi-resolution cost volume fusion, and local structure entropy optimization module work together to significantly improve the accuracy, robustness, and generalization ability of the model.

## Figures and Tables

**Table 1:** Comparing inference speed (frames per second) and parameter count (M) against PSMNet and GwcNet. (“↓” represents “descend” or “decrease”, and “↑” represents “ascend” or “increase”.)

Model	EPE ↓	FPS ↑	Parameters ↓
PSMNet	0.83	13.5	12.6 M
CIAM-T	0.73	28.4	7.8 M

**Table 2:** Performance of different methods on KITTI2012, KITTI2015, and Middlebury data sets.

Method	KITTI2012	KITTI2015	Middlebury
PSMNet	16.3	15.1	25.1
GwcNet	11.7	12.8	24.1
GANet	10.1	11.7	20.3
CoEx	7.6	7.2	14.5
DSMNet	6.2	6.5	13.8
CGI-Stereo	6.0	5.8	13.5
CFNet	5.1	6.0	19.5
Graft-PSMNet	4.3	4.8	9.7
ITSA	4.2	4.7	10.4
RAFT-Stereo	-	5.7	12.6
Ours	4.01	3.61	9.94

**Table 3:** Test results of KITTI2015 and KITTI2012 data sets.

KITTI2015 Data sets test results					
Error		D1-bg	D1-fg	D1-all	
All/All		2.56	4.84	3.61	
All/Est		2.56	4.84	3.61	
Noc/All		1.53	3.72	2.09	
Noc/Est		1.53	3.72	2.09	
KITTI2012 Data sets test results					
Error	Out-Noc	Out-All		Avg-Noc	Avg-All
2 pixels	4.27%	4.98%		0.5 px	0.5 px
3 pixels	4.01%	4.52%		0.5 px	0.5 px
4 pixels	3.64%	3.89%		0.5 px	0.5 px
5 pixels	2.80%	3.05%		0.5 px	0.5 px

**Table 4:** Ablation experiment results of different models.

Method	CIAM-T	MRCV-F	LSE	EPE(px)	D1(%)
Baseline (GwcNet)	×	×	×	0.8306	0.0279
CIAM-T	√	×	×	0.7366	0.0252
MRCV-F	×	√	×	0.7866	0.0219
LSE	×	×	√	0.6323	0.0208
CIAM-T + MRCV-F + LSE (Ours)	√	√	√	0.5878	0.0193

**Table 5:** FLOPs and parameter counts of different transformer variants.

Method	FLOPs (G)	Memory (GB)	Speed (FPS)	EPE (px)
3DConv	45.7	8.2	15.3	0.8721
ViT	62.3	11.5	12.1	0.8142
CIAM-T	58.9	9.8	18.7	0.7366
MRCV-F	72.4	13.1	16.2	0.7866

## Data Availability

The data sets generated or analyzed during this study are available from the corresponding author upon reasonable request.

## References

[1] Dosovitskiy A., Beyer L., Fischer P., Sumer S. An Image is Worth 16 × 16 Words: Transformers for Image Recognition at Scale. Proceedings of the International Conference on Learning Representations.

[2] Poggi M., Matteucci M., Fusiello A. Attention-Based Stereo Matching with Occlusion Handling. Proceedings of the Conference on Computer Vision and Pattern Recognition.

[3] Zhang Z., Liu Y., Wang H. Swin Transformer for Stereo Matching. Proceedings of the European Conference on Computer Vision.

[4] Chen Z., Xie X., Liu W., Zhang L. Dynamic Attention Cost Volume Refinement for Stereo Matching. Proceedings of the IEEE/CVF Conference on Computer Vision and Pattern Recognition.

[5] Guo K., Chen L., Wang X. Deformable Cross-Attention for Efficient Stereo Matching. Proceedings of the IEEE/CVF Conference on Computer Vision and Pattern Recognition.

[6] Wang Y., Sun W., Zhao X. (2023). Multi-Head Attention-Based Stereo Matching for Dynamic Scenes. IEEE Robot. Autom. Lett..

[7] Liu J., Sun M. Cross-Domain Attention for Dynamic Stereo Matching. Proceedings of the Thirty-Sixth Annual Conference on Neural Information Processing Systems.

[8] Zhao X., Li Y., Liu Z. (2022). Cross-Attention Guided Disparity Estimation. Pattern Recognit. Lett..

[9] Huang R., Zhang L., Liu Z. Hybrid Transformer-CNN for Enhanced Stereo Matching. Proceedings of the Thirty-Fifth Conference on Neural Information Processing Systems (NeurIPS 2021).

[10] Sun C., Xu H., Liu W. Global-Local Attention Fusion for Stereo Matching. Proceedings of the IEEE/CVF International Conference on Computer Vision (ICCV) 2021.

[11] Kendall A., Grimes M., Garnett R. End-to-End Learning of Geometry and Context for Deep Stereo Regression. Proceedings of the IEEE/CVF Conference on Computer Vision and Pattern Recognition (CVPR) 2020.

[12] Chang J., Chen Y. PSMNet: Pyramid Stereo Matching Network. Proceedings of the IEEE/CVF Conference on Computer Vision and Pattern Recognition (CVPR) 2021.

[13] Zhang Q., Yang Z., Chen J. Cascade Cost Volume Construction for Stereo Matching. Proceedings of the Conference on Neural Information Processing Systems (NeurIPS) 2020.

[14] Huang H., Zhang Y. (2024). CostVolume Transformer: A Transformer-Based Stereo Matching Framework. IEEE Trans. Pattern Anal. Mach. Intell..

[15] Tang T., Li S. Adaptive Entropy Weighting for Low-Texture Disparity Estimation. Proceedings of the 2023 International Conference on Computer Vision ICCV 2023.

[16] Sun J., Liu M. GNN-CostVol: Graph Neural Network-Based Cost Volume Refinement for Occlusion Handling. Proceedings of the IEEE/CVF Conference on Computer Vision and Pattern Recognition (CVPR) 2023.

[17] Guo X., Huang Y., Li C. Group-Wise Correlation for Cost Volume Refinement. Proceedings of the European Conference on Computer Vision (ECCV) 2022.

[18] Liu J., Zhang Q., Chen L. (2023). Hybrid Optimization for Stereo Matching. IEEE Trans. Pattern Anal. Mach. Intell..

[19] Xu L., Liu S., Tang L. Attention-Guided Cost Aggregation for Stereo Matching. Proceedings of the International Conference on Computer Vision (ICCV) 2022.

[20] Zhao Z., Wang Q. Multi-Task Stereo Matching with Semantic Segmentation Integration. Proceedings of the European Conference on Computer Vision (ECCV) 2022.

[21] Chang T., Yang X., Zhang T., Wang M. Domain Generalized Stereo Matching via Hierarchical Visual Transformation. Proceedings of the IEEE/CVF Conference on Computer Vision and Pattern Recognition (CVPR) 2023.

[22] Chen C., Zhang Y. Cycle-Consistent Contrastive Learning for Self-Supervised Domain Adaptation. Proceedings of the International Conference on Computer Vision (ICCV) 2023.

[23] Fang F., Wang L. MAML-Adapt: Few-Shot Meta-Learning for Domain Generalization in Stereo Matching. Proceedings of the The Thirty-Sixth Annual Conference on Neural Information Processing Systems (NeurIPS 2022).

[24] Lin H., Zhang R. DisparityGAN: Generating Synthetic Disparity Maps for Generalization Enhancement. Proceedings of the IEEE/CVF Conference on Computer Vision and Pattern Recognition (CVPR) 2024.

[25] Xu H., Zhang W. (2020). Local Structural Regularization in Stereo Matching. Int. J. Comput. Vis..

[26] Yang F., Zhang X., Li J. Hierarchical Stereo Matching Network. Proceedings of the IEEE/CVF Conference on Computer Vision and Pattern Recognition (CVPR) 2021.

[27] Lin K., Zhang R. Contrastive Learning for Domain Generalization in Stereo Matching. Proceedings of the Thirty-Sixth Annual Conference on Neural Information Processing Systems (NeurIPS) 2022.

[28] Wang H., Liu Y. Entropy-Guided Pyramid Fusion for Global-Local Synergy. Proceedings of the IEEE/CVF Conference on Computer Vision and Pattern Recognition (CVPR) 2023.

[29] Li J., Chen T. Entropy-Regularized Contrastive Learning for Unsupervised Domain Adaptation. Proceedings of the 38th Annual AAAI Conference on Artificial Intelligence (AAAI-2024).

[30] Tang S., Zhang W., Chen J. Local Structural Entropy for Stereo Matching. Proceedings of the International Conference on Computer Vision (ICCV) 2023.

[31] Li X., Wang X., Zhang Y. Entropy-Weighted Multi-Scale Fusion for Stereo Disparity Estimation. Proceedings of the IEEE/CVF Conference on Computer Vision and Pattern Recognition (CVPR) 2021.

[32] Tzeng E., Hoffman J., Saenko K. Adversarial Discriminative Domain Adaptation. Proceedings of the European Conference on Computer Vision (ECCV) 2020.

[33] Zhang T., Liu Z., Wang H. Domain-Invariant Feature Extraction for Stereo Matching. Proceedings of the Conference on Neural Information Processing Systems (NeurIPS) 2021.

[34] Wang F., Zhang Y., Zhou Z. Meta-Learning for Cross-Domain Stereo Matching. Proceedings of the International Conference on Learning Representations (ICLR) 2023.

[35] Fang F., Wang L. MAML-Adapt: Efficient Meta-Learning for Domain Generalization. Proceedings of the Thirty-Sixth Annual Conference on Neural Information Processing Systems (NeurIPS 2022).

[36] Lin H., Zhang R. DisparityGAN: Generating Synthetic Data for Cross-Domain Generalization. Proceedings of the IEEE/CVF Conference on Computer Vision and Pattern Recognition (CVPR) 2024.

[37] Lin J., Chen M., Wang X. (2024). Generative Adversarial Networks for Cross-Domain Stereo Matching. IEEE Trans. Pattern Anal. Mach. Intell..

[38] Huang Z., Yang H. Domain Generalized Stereo Networks Using Contrastive Learning. Proceedings of the European Conference on Computer Vision (ECCV) 2022.

[39] Zhao Q., Li X., Zhang Z. Unsupervised Domain Adaptation for Stereo Matching. Proceedings of the IEEE/CVF International Conference on Computer Vision (ICCV) 2022.

[40] Chang Y., Chen Z. (2018). Local Context Enhancement for Stereo Matching via Dilated Convolutions. Int. J. Robot. Res..

[41] Hinton G.E., Osindero S., Teh Y.W. (2006). A fast learning algorithm for deep belief nets. Adv. Neural Inf. Process. Syst..

[42] Bishop C.M. (2006). Global entropy minimization. Pattern Recognition and Machine Learning.

